# Opportunities and limits of controlled-environment plant phenotyping for climate response traits

**DOI:** 10.1007/s00122-021-03892-1

**Published:** 2021-07-24

**Authors:** Anna Langstroff, Marc C. Heuermann, Andreas Stahl, Astrid Junker

**Affiliations:** 1 Department of Plant Breeding, IFZ Research Centre for Biosystems, Land Use and Nutrition, Justus Liebig University Giessen, Heinrich Buff-Ring 26, 35392 Giessen, Germany; 2 Leibniz Institute of Plant Genetics and Crop Plant Research (IPK) Gatersleben, Corrensstr. 3, OT Gatersleben, 06466 Seeland, Germany; 3 Institute for Resistance Research and Stress Tolerance, Federal Research Centre for Cultivated Plants, Julius Kühn-Institut (JKI), Erwin-Baur-Strasse 27, 06484 Quedlinburg, Germany

## Abstract

Rising temperatures and changing precipitation patterns will affect agricultural production substantially, exposing crops to extended and more intense periods of stress. Therefore, breeding of varieties adapted to the constantly changing conditions is pivotal to enable a quantitatively and qualitatively adequate crop production despite the negative effects of climate change. As it is not yet possible to select for adaptation to future climate scenarios in the field, simulations of future conditions in controlled-environment (CE) phenotyping facilities contribute to the understanding of the plant response to special stress conditions and help breeders to select ideal genotypes which cope with future conditions. CE phenotyping facilities enable the collection of traits that are not easy to measure under field conditions and the assessment of a plant‘s phenotype under repeatable, clearly defined environmental conditions using automated, non-invasive, high-throughput methods. However, extrapolation and translation of results obtained under controlled environments to field environments is ambiguous. This review outlines the opportunities and challenges of phenotyping approaches under controlled environments complementary to conventional field trials. It gives an overview on general principles and introduces existing phenotyping facilities that take up the challenge of obtaining reliable and robust phenotypic data on climate response traits to support breeding of climate-adapted crops.

## Introduction

The increase in atmospheric CO_2_ leads to rising temperatures and changing precipitation patterns (IPCC 2014), which have a strong impact on agricultural production. While the anticipated effects of climate change may be beneficial for the growing conditions in some regions (e.g. boreal region), climate change may cause extended or more intense periods of stress and different combinations of stress conditions in other regions (Lobell et al. 2011; Asseng et al. 2015; Leng and Hall 2019; Ray et al. 2019). The anticipated climate change affects crops in their environment in multiple ways. On the one hand, elevated atmospheric CO_2_ concentration stimulates photosynthesis and may benefit shoot growth and yield production as well as abiotic stress adaptation by enhanced root growth, decreased stomatal conductance, and, thus, improved water use efficiency (WUE) (Lopes and Foyer 2011). On the other hand, it is most likely that these positive effects caused by an increased CO_2_ concentration will be negated by increasing temperature and changes in precipitation patterns (Lobell and Gourdji 2012). Global warming shifts the timing of seasons and thus the phenology of plants: leaf development and flowering begins earlier in the year (Piao et al. 2019; Menzel et al. 2020). The extension of the frost-free period can be advantageous in certain regions, but may also lead to heat stress and summer droughts in other regions (Trnka et al. 2011; Teixeira et al. 2013), by shifting the vegetation period, which increases evaporation through plant growth in spring (Lian et al. 2020). Drought stress promotes a diverse set of reactions of the plant that vary with plant size and the intensity and timing of the drought (Tardieu et al. 2018). Plants exposed to drought conditions will have an inhibited photosynthesis by stomata closure and reduced leaf area, leading to reduced yields (Blum 1996). Water deficit, heat stress or combinations thereof during the reproductive development of the plant affect pollen viability, fertility, and seed set, resulting in major yield losses (Dong et al. 2017; Mahrookashani et al. 2017; Fábián et al. 2019). All these effects are summarizable as the environmental factor (E). On the contrary, adjustments in tilling, sowing, irrigation, application of fertilizer and plant protection, summarizable as the management factor (M), and modification of the genotype of the cultivated crops through breeding, as the genetic factor (G), are called upon to diminish negative impacts of climate change effects on crop production (Challinor et al. 2014).

The phenotype (P) is a single trait or cumulative information that represents just one single constellation of the total sum of possible phenotypes, referred to as the phenome (Davis 1949). Given the near infinite combinations (G x E x M) between a genotype (G), its environment (E) and crop management (M), the very most import question is to find optimal G x M combinations that enables under given future E the best P, in terms of qualitative and quantitative outcome of crop production. Following the assumptions of the climate forecasts, future varieties will have to be significantly more tolerant against diverse stresses. Breeding driven adaptation of crops, as a main pillar of measures to counter negative effects of climate change, requires the identification of genetic variation contributing to an enhanced adaptation to abiotic stress as well as the selection and evaluation of performance under climate conditions of the expected future target environment. In case that breeders realize an insufficient genetic diversity for climate-adaptation traits within their genepool, an extension of the genetic resources might bring advantages. Elite cultivars from regions that are usually warmer and drier, and are already exposed to predicted future climates of the target region, represent an interesting source of genetic variation in order to breed cultivars better adapted to future climate conditions (Reynolds et al. 2015; Atlin et al. 2017). Due to the high environmental interaction (e.g. with the length of days), a transfer to completely different regions may not be as straightforward. In addition, it must be considered that despite rising temperatures and dry vegetation periods, "old" traits remain relevant (e.g. winter hardiness). Therefore, it is not spared to better elicit the exact adaptation mechanisms and disentangle their genetic determinants. Importantly, questions need to be answered about which traits are particularly relevant and, more importantly, how they interact with each other under future climate conditions. If the infinite number of G x E x M interactions are considered, it becomes clear that answers cannot be given based on experiments alone, but knowledge must also be acquired with the help of models (Cooper et al. 2021a). The training of these models requires most realistic and reliable phenotypic data. Even though phenotypic data which originates from field experiments are undoubtedly preferred as they describe the crops in their “real” target environment, most likely several questions are not to be answered without the methodological bridge of CE phenotyping.

## To control or not to control?—That is the question

Phenotyping for climate response traits requires clearly defined environmental conditions corresponding to the experimental question. Field grown crops are unsheltered from biotic and abiotic stresses, seasons, weather extremes (short-term condition of a magnitude exceeding a predefined threshold, e.g. heat wave), and climate change (long-term alteration of average weather patterns), which challenge plant performance and yield (Lobell and Field 2007). Field environments are characterized by strong dynamics in light intensity and quality, air and soil temperatures, wind exposure, water and nutrient supply and soil compaction (Poorter et al. 2016), leading to a high variability of environmental conditions which complicate the evaluation and interpretation of phenotypic data from field trials. In extreme cases, highly varying environmental conditions within two or more years can weaken the expected year-to-year correlation considerably: a meta-study has shown that the year-to-year correlation of yield data can be very low (*r*^*2*^ = 0.08; Poorter et al. 2016). The rationale for CE phenotyping comes for three major reasons.

First, future scenarios cannot be realized in the field today and require defined, repeatable and deliberately manipulable environmental growth conditions. This concerns not only tests in breeding activities of existing crop species but also the evaluation of possible other species that will become relevant in the future in latitudes where they are not grown today.

Second, while some traits are relatively easy to phenotype under field conditions (e.g. vegetation indices), some are hardly phenotypable in the field or only at the cost of high labour intensity. Thus, for several traits non-destructive phenotyping under CE allows significant improvements in precision and enables tracking the course of growth (e.g. root morphology and recording of diurnal transpiration profiles within days and across the lifecycle).

Third, accurate phenotyping based on standardization of a certain E x M constellation is pivotal to reduce the residual error of experiments and enables a reliable heritability estimation, which is a key element of breeding gain (Lush 1943). CE facilitates a reduction of environmentally induced variation, which is also required for molecular phenotyping, in order to gain profound understanding of the interplay of genomic, transcriptomic, proteomic, and metabolic processes in the plant.

Despite the benefits of CE phenotyping, it should not be considered as a substitute for field phenotyping but rather as a complementing tool that allows to gain insights that are hard to obtain under field conditions but are essential to receive a mechanistic understanding of pathways and/or enable the training of models, provided a well-conceived experimental setup is implemented. Especially when complex multigenic traits that show a high interaction with environmental factors are studied, it is always necessary to ensure that findings originating from controlled-environment experiments are significant in the target environment. The beforehand mentioned meta-study compared phenotypic data obtained from controlled environments with phenotypic data from field trials and found only a low correlation between lab and field conditions (Poorter et al. 2016). Reasons for this poor comparability are manifold: lower light intensities and higher temperatures, especially in early developmental stages, and often plant densities in controlled-environment scenarios diverging from what the plants experience in field conditions which ultimately affect total plant biomass, growth rates, leaf area and plant architecture (Poorter et al. 2012). Furthermore, plants grown in CE are regularly constrained by pot size and thus reduced soil volume, which impedes root growth, affects biomass production and the plants reaction to water and nutrient availability (Poorter et al. 2012; Passioura 2006), making it especially difficult to phenotype for climate response traits. To overcome the poor transferability of results from controlled to field conditions, CE phenotyping facilities offer the possibility to approximate the field as closely as possible by generally refining standard growth parameters e.g. by adjusting light and temperature conditions to nature (Song et al. 2018), due to regular feedback irrigation (Gosa et al. 2019), by providing a sufficient root volume by using larger pots (Hohmann et al. 2016), or by a combination of them (Stahl et al. 2020). This allows comprehensive phenotyping of plant responses to different applied stress scenarios without losing too much relevance of the acquired phenotypic data to field environments. Increasing temperatures in two successive stages from 15 to 25 °C over the time of maize development, simulating spring temperatures of a temperate region, substantially improved rank correlations between biomass data of the glasshouse cultivation and of field trials (Junker et al. 2015). Furthermore, several reports gave evidence for the potential of artificially fluctuating light to mimic naturally occurring photosynthetic acclimation processes (Suorsa et al. 2012; Hirth et al. 2013). Chiang et al. (2020) examined the effect of different patterns of light, temperature and humidity (fixed day and night conditions, conditions following a sinusoidal curve, and mimicking of records of environmental conditions in field trials) in an indoor facility on different biomass, pigmentation and leaf gas exchange parameters in a range of plant species, comparing them to field grown plants. Depending on the species and examined parameter, plants exposed to the sinusoidal conditions or mimicked field conditions were more similar to the field-grown plants compared to the plants exposed to fixed day and night temperatures (Chiang et al. 2020), showing the relevance of short-term fluctuations of environmental conditions, when a higher lab-to-field comparability is desired.

The question may arise in which part of the process for breeding climate-adapted crops CE phenotyping offers a particular added value. As one area of application, CE can be considered in the context of pre-breeding to screen potential crossing parents and to identify donors of certain traits of interest. For selection of entire breeding populations, CE can supplement large-scale field trials in order to gain additional precise phenotypic data that can serve as a training dataset for crop models. Those can significantly understand responses across different scales and quantify trade-off effects of manipulation of certain pathways (Wu et al. 2019). Moreover, very recently Cooper et al. (2021b) illustrated opportunities to integrate mechanistic crop models with quantitative genetics to enhance selection response. In another sense, CE phenotyping can also be applied in one of the final stages of the breeding cycles in order to thoroughly evaluate responses to specific environmental conditions (Ghanem et al. 2015). However, this multi-tier phenotyping strategy, with increasing trait resolution and decreasing genotype number in each subsequent phenotyping stage, can be rather seen as an evaluation tool as potential genotypes of interest might be already lost in early selection stages.

## State-of-the-art phenotyping

In order to deal with the strong dynamics in environmental conditions in field environments, a diverse set of techniques and facilities were developed and allowed studying the phenotypic response of a plant to a certain environment.

A wide range of facility types exists that allow a varying level of environmental control and increasing options for collection of phenotyping in an unprecedented multi-faced resolution. These range from basic facilities, such as rainout shelters, that enable control over one or a few factors, to greenhouses that can influence several environmental factors up to fully controlled facilities that simulate certain field-like environmental scenarios.

Phenotyping of dynamic field environments is mainly performed by cameras and sensors mounted to movable carrier platforms, which are exposed to potentially greater interference from environmental variables during the measurement procedure (e.g. intensity of sunshine, wind, etc.) than indoor systems. With regard to comparability of different measurement runs between locations and time points, unfavourable environmental variables like strong windfall moving leaves and whole plant canopies, or clouds blocking direct sunlight and thereby causing colour distortions during imaging can render field phenotyping inferior to indoor phenotyping under controlled environments in differentiating between the contribution of genotypic variation to a recorded image of a phenotype from noise introduced by the environment. Despite this susceptibility, field phenotyping technologies generate meaningful data and contribute significantly to the understanding of a crop interacting with its environment (Araus and Cairns 2014).

Non-invasive high-throughput phenotyping of field stands is realized by camera systems mounted to mobile tractors (Salas Fernandez et al. 2017) or unmanned aerial vehicles, which score plant height and extract colour-related traits from crop canopies with high repeatability and over all cover greater areas in shorter time (Madec et al. 2017; Yang et al. 2017). Potentially highest throughput for traits like normalized difference vegetation index could be achieved using satellite images, which capture a whole field within one picture, and can lead to highest correlation with biomass and yield compared to other techniques (Tattaris et al. 2016). Field phenotyping with higher resolution can be achieved by stationary equipment built around a field stand. At the Rothamsted Research facility, UK, a fixed crane equipped with cameras was successfully used to determine wheat heading and flowering stages by high-throughput phenotyping a field stand (Sadeghi-Tehran et al. 2017). A low level of control can be applied by automatic rainout shelters to field stands and the phenotype as a response to water depletion can be recorded (Beauchêne et al. 2019). Any of the above-mentioned field phenotyping devices provide a smart solution to dissect G x E x M interaction, but all are confined to the environment present at the point in time and space of the respective measurement (Araus and Cairns 2014). Furthermore, field experiments cannot be performed under future environmental conditions predicted to be prevalent at a certain location like higher average temperatures due to climate change (Pan et al. 2015). Higher minimum temperatures, especially higher night-time temperatures, do negatively impact yield development (Prasad et al. 2008, Hatfield et al. 2015). Artificially increasing night-time temperature by covering small areas with meshes achieved only a slight increase and it was only applicable for small research field sites (Beier et al. 2004).

**Fig. 1:**
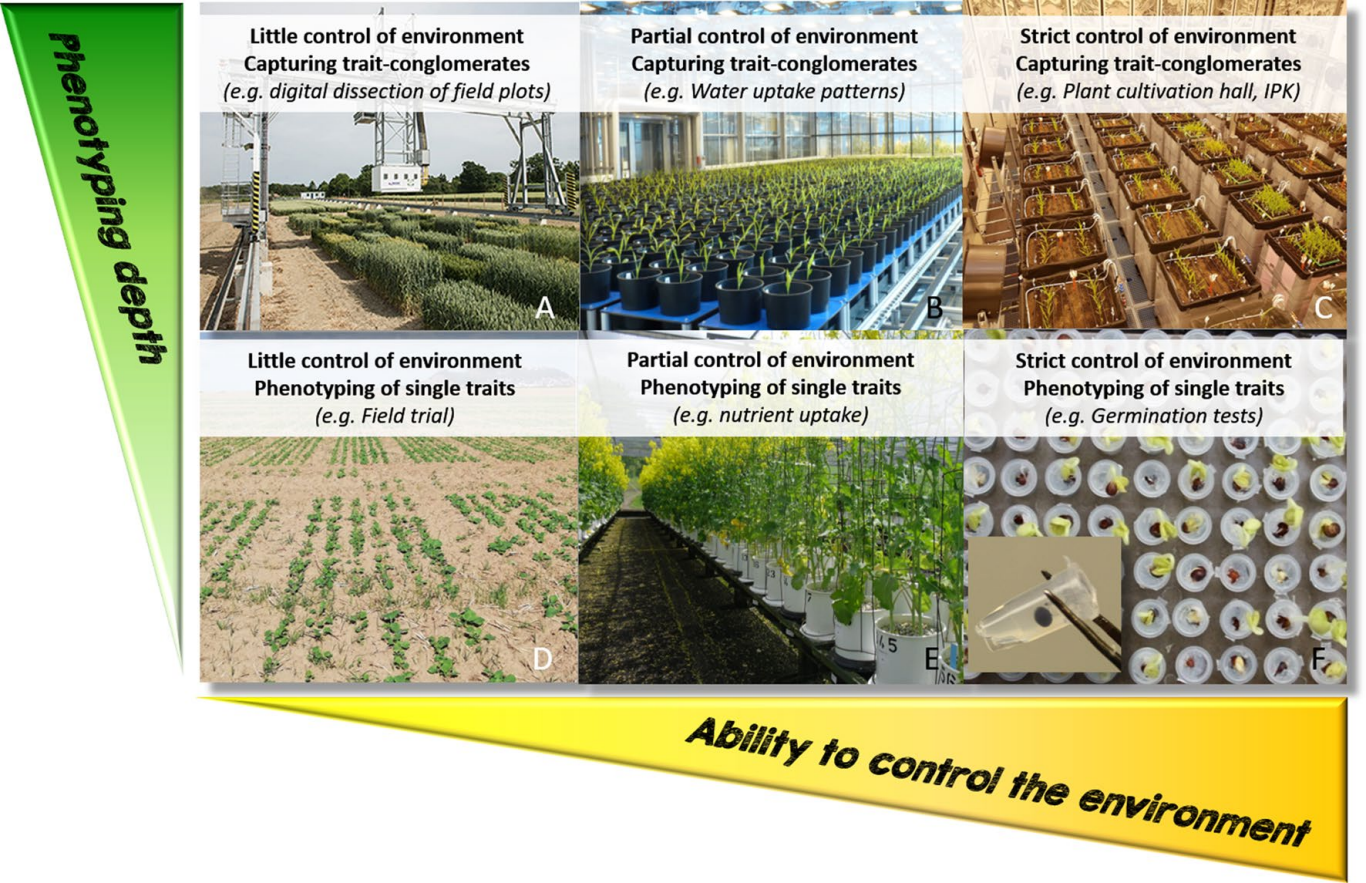
Illustration of the different phenotyping depths for phenotyping of trait complexes (vertical axis) and degree of environmental control (horizontal axis) using exemplary facilities for different facility categories. Picture sources: **a** Sadeghi-Tehran et al. (2017), **b** Junker et al. (2015), **c–f** own records

Indoor phenotyping facilities limit the randomness of environmental variation and implement a certain degree of control but increase the artificiality of the growth conditions (Fig. 1). More control and thereby higher repeatability are achieved by integrating high-throughput phenotyping systems into indoor glasshouses and growth chambers. There are many approaches to improve phenotyping under partially controlled environments (Yang et al. 2020; Pieruschka and Schurr 2019). Advances in sensor technology paved the way for the development of high-throughput-phenotyping platforms that provide a broad applicability for phenotyping under both controlled and field conditions (Sadeghi-Tehran et al. 2017; Czedik‐Eysenberg et al. 2018; Maes and Steppe 2019).

### Drought and salt stress

High-throughput shoot phenotyping facilities were integrated into glasshouses all around the globe following the same concept of transporting the plants to imaging stations (Plant-to-sensor), which are equipped with cameras that cover different spectra of the electromagnetic radiation ranging from visible light to near infrared, that capture images of all major crop species like rice (Hairmansis et al. 2014; Yang et al. 2014), sorghum (Neilson et al. 2015), maize (Muraya et al. 2017; Zhang et al. 2017), barley (Chen et al. 2014; Neumann et al. 2015), and canola (Knoch et al. 2019). This architecture allows glasshouses to be built large enough to phenotype crop species on a whole population level and associate natural genomic variation with image-derived traits in a genome-wide association study e.g. in rice (Yang et al. 2014), in maize (Muraya et al. 2017), and also in canola (Knoch et al. 2019). Except from light, glasshouses shield the growth area from precipitation and outside temperature dynamics, which makes the application of abiotic stresses like water or nitrogen deficiency and increased soil salinity straight forward (Chen et al. 2014; Hairmansis et al. 2014; Neilson et al. 2015; Neumann et al. 2015). During applied drought stress, plants were phenotyped non-invasively in a daily interval and wilting, the inflection point at rewatering, and the recovery phase could all be modelled and closely tracked (Chen et al. 2014). The heritability of some traits decreased during drought and recovered after rewatering, while others were highly heritable throughout the experiment, indicating that having a multitude of traits is beneficial to precisely characterize a phenotype with all its experiment specific characteristics (Chen et al. 2014). While the increase in global mean temperature is a consensus between studies (Gornall et al. 2010; Pan et al. 2015), its impact on agriculture and severity of drought is a complex topic to predict on a global scale (Gornall et al. 2010). Robust drought indices taking meteorological, agricultural, and socioeconomic droughts into account are necessary to characterize and predict the severity of droughts (Mukherjee et al. 2018) as crop yield of major crop species is predicted to change differently in response to drought and locality of the regions they are grown in (Leng and Hall 2019). The increase of soil salinity due to sea-level rise in coastal regions (Dasgupta et al. 2015) and due to prolonged droughts and decline in precipitation in arid and semi-arid regions (Corwin 2020) further impairs crop yield under a global warming scenario. Controlled-environment phenotyping can help to elucidate the G x E interaction under well-defined drought conditions (Chen et al. 2014; Neilson et al. 2015; Neumann et al. 2015) and soil salinity levels (Hairmansis et al. 2014). Large volume containers can counter the constraint of typical CE pot size (Hohmann et al. 2016; Thomas et al. 2018; Stahl et al. 2020).

### Temperature

The global temperature increase itself has one of the most drastic effects on yield development, and without taking CO_2_-fertilization or further crop improvement into account, each degree-Celsius increase is predicted to decrease crop yield on average between three and seven percent depending on the species (Zhao et al. 2017). Experiments under controlled environments with increased mean temperature found that elevated temperature during the onset of the reproductive stage of development in maize reduced crop yield by up to 90% but increased vegetative biomass overall (Hatfield et al. 2015). The response of crop species differs with regard to yield development. The combination of different crop species grown at several locations in Australia allowed the deduction of sensitivity patterns against environmental factors (Dreccer et al. 2018). Hence, the temperature responses of crops during certain developmental stages are unique in their degree of impact and in their direction (Luo 2011). In Arabidopsis, the genetic architecture of a developmental stage-specific response to temperature extremes was dissected in a GWAS and found to differ between pre- and post-anthesis in a controlled-environment experiment (Bac-Molenaar et al. 2015).

Combining CE and repeated non-invasive high-throughput phenotyping enabled the detection of time resolving quantitative trait loci (QTL) action of alleles in maize (Muraya et al. 2017), in canola (Knoch et al. 2019), and in Arabidopsis (Meyer et al. 2020). The occurrence of growth stage-specific QTL during GWAS highlights the importance of repeated phenotyping in addition to endpoint yield phenotyping to gain knowledge in growth dynamics and the underlying genetic architecture, which leads to the final phenotype. CE phenotyping could provide insight into the genetic architecture of developmental stage-specific temperature responses, to help stakeholders breed climate change resilient crops. For stakeholders with lower budgets, the hurdle of entry into CE phenotyping was lowered significantly by recent technology advancements. Any greenhouse can be upgraded into a high-throughput phenotyping platform using affordable Raspberry Pi computers (Minervini et al. 2017), an approach which generates high-quality data sufficient for deep neural networks (Samiei et al. 2020).

Although most of the CE phenotyping experiments were run under constant conditions with at most a mild temperature shift only between night and day, a controlled environment does not necessarily equal constant climate conditions. While climate change will affect the whole ecosphere of the planet, lower or higher latitudes will be differently affected (Pan et al. 2015). Lower latitudes, and thereby crop species currently adapted to those regions, will be more severely afflicted than high- and especially mid latitudes (Rosenzweig et al. 2014). Flowering time of *A. thaliana* accessions was assessed by simulating the dynamics of the daily temperature, day length, and the average light intensity per day in a growth chamber simulating both spring and summer in Sweden and Spain to dissect the genetic components leading to acclimation processes between the latitude of origin (Li et al. 2010). Including the dynamics of an environment into CE phenotyping can shed light onto understanding the genetic differences of acclimation processes between varieties of the same species to specific latitudes. In addition, CEs allow species from other regions to be tested for introduction into a target environment through targeted simulation.

In general, elevating the levels of total intercepted light from common growth chambers to levels found in a field environment can lead to a 60% increase in biomass accumulation (Poorter et al. 2016). Furthermore, a 10 °C difference in temperature during the growth period amounts to a difference of accumulated biomass to up to 600%, while natural diurnal temperature shifts caused by day night cycles and seasons are commonly neglected in controlled environments, which is an additional explanation for the poor lab-to-field correlation between phenotyping experiments (Poorter et al. 2016). High-throughput phenotyping needs to be combined with dynamic environments to dissect genetic variation under natural or field-like environmental dynamics, but still in a controlled and repeatable fashion to not suffer from the randomness of field experiments. Bao et al. (2019) approach this discrepancy by using an array of eight precisely controlled growth chambers, which are phenotyped by a mobile robotic rover. The rover is equipped with a matrix of imaging sensors covering different spectra and drives autonomously into the environment chambers (Sensor-to-plant), which themselves are designed to achieve field-relevant dynamics in temperature, humidity, light intensity, and also to simulate a range of CO_2_ concentrations (Bao et al. 2019). However, the chambers together cover a growth area of only 14.4 m^2^ which requires researchers to invest more into prior experimental planning to achieve interpretable results.

### Carbon fertilization

Current and future climate change will further increase global temperatures accompanied by increased CO_2_ levels due to anthropogenic carbon emissions (Pan et al. 2015). Carbon fertilization due to elevated CO_2_ levels is expected to alleviate the negative impact of temperature increase on yield development, with a stronger benefit for C3 than C4 plants (Fuhrer 2003; Pongratz et al. 2012). The effects of elevated CO_2_ levels on plant growth are not expected to act ubiquitously positive but depend in their amplitude on the interaction with regional environments (McGrath and Lobell 2013), on the frequency of droughts (Jin et al. 2018), and will likely decrease the nutritional value of both crops (Taub et al. 2008) and vegetables (Dong et al. 2018). A large-scale comparison of yield increases under elevated CO_2_ concentrations found 50% lower yield increase for C3 plants grown in the field than indoor and no increase for C4 plants (Long 2006). High-throughput phenotyping platforms should be able to simulate dynamic environments, as just increasing CO_2_ levels but keeping other environmental factors steady will not reflect the full complexity of G x E interaction necessary to generate high-quality input parameters for modelling plant performance under predicted climate change conditions.

### Simulated field

**Fig. 2:**
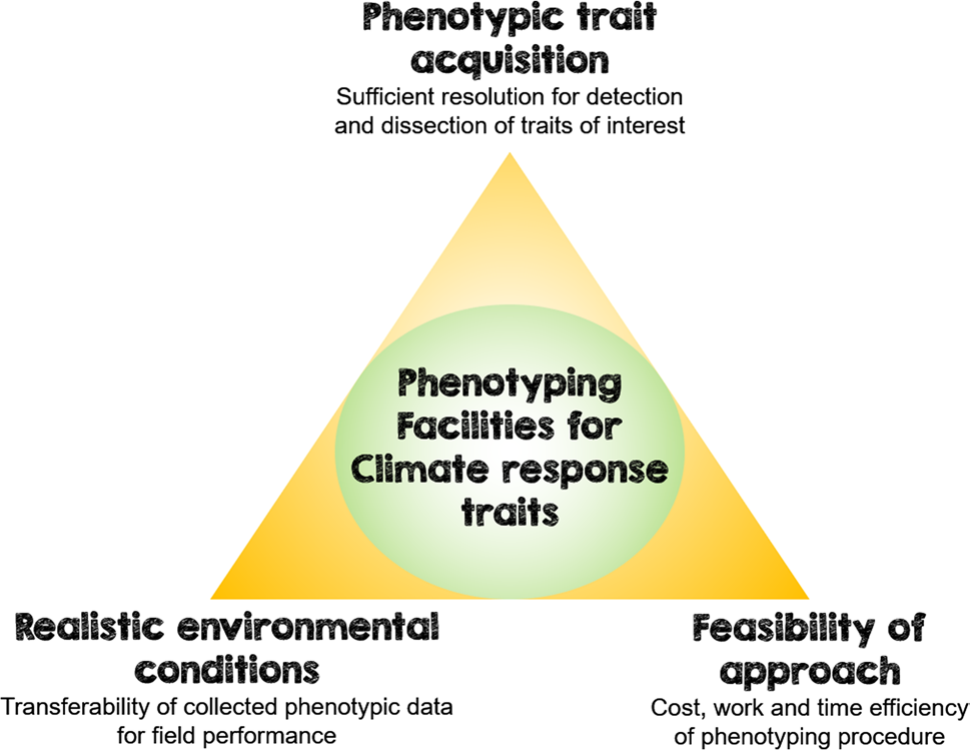
The circle of phenotyping methods within the triangle of conflicting objectives illustrates that improvement of phenotyping facilities in one aspect comes at the expense of the others

CE phenotyping facilities have contributed to advances in knowledge in various areas with their individual strengths. Still, it turns out that each of the mentioned approaches is mostly not ideal in all requirements of phenotyping. Some facilities allow a multifaceted data acquisition in a level of detail not achieved so far but may have weaknesses with regard to the comparability of plant growth conditions. On the other hand, some systems show their special strengths in the cultivation of plants in a field-like manner but may not be equipped with high-resolution sensors. The phenotyping of adaptation traits in the context of climate change requires a triad of realistic growth conditions, sufficient resolution of relevant trait detection, and financial and temporal feasibility of the investigations—in other words “Breeders Friendly” (Reynolds et al. 2020). Depending on the objective of the study, the comparative advantages of one system may, to a certain extent, justify the neglect of another property (Fig. 2). To bridge the gap between controlled environments and field phenotyping, a unique plant growth and high-throughput phenotyping facility, is currently in the prototype phase at the IPK Gatersleben (personal communication Altmann T. 2020). It is designed like a huge growth chamber, shielded from the outside environment, but recreating field-like dynamics of temperature, light quality and quantity, relative air humidity, wind simulation, and the dynamics of CO_2_ from ambient to up to 1200 ppm. Plants are grown in containers filled with layers of soil, loess, and gravel, which are large enough to mimic a plot of crops commonly found in field stands. An isothermal climate of up to six meters from the top soil levels allows the cultivation of any major crop species like barley, oilseed rape or maize.

Installations and technologies in this review represent only a fraction of the available phenotypic resources as noted by Yang et al. (2020) who counted 82 mechanized phenotyping platforms across the globe. Advances in phenotyping technologies, in- and outdoors, have been a joint effort of researchers with backgrounds in multiple disciplines from many countries. The IPPN, EPPN, and DPPN associations act as resources to increase the visibility of the available phenotypic resources in the world (https://www.plant-phenotyping.org/infrastructure_map, https://eppn2020.plant-phenotyping.eu/EPPN2020_installations, https://dppn.plant-phenotyping-network.de/index.php).

## Automated digital trait assessment

The above-listed controlled-environment phenotyping facilities offer particular advances for phenotyping by enabling automated high-throughput phenotyping with a lower intensity of manual labour, higher accuracy, “stress on demand”-conditions, and a higher frequency of time points for assessing traits over the crop lifecycle. Besides the determination of a suitable and well-designed experimental set-up, it is also necessary to consider which traits are relevant in order to identify the performance of the plant in unfavourable environments (Negin and Moshelion 2017). A number of phenotypic parameters are available that are related to the plant’s tolerance against several stress conditions, are robust and reliable, show a higher heritability than yield or yield stability, and can be used to measure plants characteristics towards drought and heat tolerance (Richards et al. 2010; Gupta et al. 2012).

Phenotyping approaches can be divided into two classes, invasive or non-invasive methods. The former needs to sample parts of a plant or harvest the whole plant, which is a destructive process and prevents any successive measurement of the same tissue or the same individual. It generates a snapshot of the characteristics of a plant, which interacted with a specific environment until the time of sampling. The latter collects information from any tissue or the whole plant by non-destructive measurements and enables subsequent recordings from the identical individual. Thereby it can generate a series of phenotypic snapshots, which are still limited by the exposure of the plant to a specific environment but are not limited by time and can track changes in the phenotype throughout the life cycle of a plant. When comparing destructive to non-destructive methods, a high correlation is achieved between shoot fresh and dry weight, destructively measured leaf area and corresponding predictions from RGB images (Ge et al. 2016).

### Image-derived trait acquisition

The most common traits which are phenotyped non-invasively in a high-throughput fashion, are the result of the interaction between a plant and photons from diverse frequencies of the electromagnetic radiation. In the visible light spectrum, red green blue (RGB) cameras are routinely used to capture the reflection of photons from the plant and extract image related traits like leaf area or digital biomass (Klukas et al. 2014; Chen et al. 2014). Digital and real biomasses are both multigenic traits, but in contrast to real biomass, digital biomass can be repeatedly measured and the temporal dynamics of the genome regions significantly linked with the measured trait at a specific time point can be dissected (Muraya et al. 2017). The reflection of photons from the near-infrared spectrum between 750 and 1400 nm in plant leaves is directly linked to water content (Seelig et al. 2008), and can be used to assess the severity of drought in controlled environments in high-throughput phenotyping platforms (Muscolo et al. 2015) or provide information about the vigour and water content of crops in the field with UAV or even satellite mounted cameras (Hunt et al. 2016; Di Gennaro et al. 2018). Fluorescence, the absorption of photons and later re-emission of photons with mostly lower energy levels, is used to detect chlorophyll fluorescence in plant leaves, which is directly linked to the maximum photosystem II (PSII) efficiency and the PSII operating efficiency (Tschiersch et al. 2017), and can be used to detect drought stress (Mathobo et al. 2017) or act as biomarkers for abiotic stress in crop phenotyping (Kalaji et al. 2018). Hyperspectral cameras, which capture the reflection of a wide range of wavelengths often from 400–1000 nm, can be used to detect abiotic plant stress like salt stress (Sytar et al. 2017) or biotic stress like scoring the disease severity of charcoal rot in soybean (Nagasubramanian et al. 2019). The high dimension of data due to scanning a continuum of the electromagnetic spectrum, which also generates a large amount of data, makes it challenging to handle the output from hyperspectral imaging, which has led to the frequent implementation of machine learning for the analysis of hyperspectral data (Lowe et al. 2017). Information from photons with a higher energy like X-ray or gamma ray radiation is used for high-resolution root phenotyping by X-ray computer tomography (CT) or positron emission tomography (PET) (Atkinson et al. 2019). Schmidt et al. (2020) recently demonstrated the potential of using an X-ray computed tomographic analysis to scan wheat ears exposed to drought and heat stress in order to evaluate grain setting and grain size incl. deformations along the ear. Given the potential to scale the throughput up to several thousands of heads the approach seems to be suitable for large-scale screenings in breeding programs. High-resolution root phenotyping and high-resolution phenotyping of plant tissues can also be performed by magnetic resonance imaging (MRI), measuring the relaxation of the spin of atomic nuclei in a magnetic field (Jahnke et al. 2009; van Dusschoten et al. 2016).

Image-derived traits capture the integrated prior interaction of environment and genotype which led to the phenotype measured at one moment in space and time, which by nature makes them multigenic traits. Due to their integrated high informational load, image-derived traits can be effectively used to not only analyse the plant stress or health status, but also to predict a possible phenotype from the observation of the performance of a genotype under a specific environment. Light reflection spectroscopy led to the prediction of forage quality in ryegrass accessions using hyperspectral imaging (Shorten et al. 2019), of pepper quality using near-infrared reflectance spectroscopy (Toledo-Martín et al. 2016), of biotic interaction of sugar beets with nematodes (Joalland et al. 2017), of abiotic interaction of rice with arsenic load in agricultural soil (Shi et al. 2016), of bean texture quality (Mendoza et al. 2018), and also whole plant biomass from digital biomass (Chen et al. 2018). A latest summary of the application of hyperspectral to investigate yield limits in wheat is given by Bruning et al. (2020).

### Transpiration and related traits

The ability to accurately measure parameters such as water uptake and transpiration helps to dissect water use efficiency and transpiration efficiency and supports breeding for drought tolerance. A parameter used for assessment of plant transpiration is carbon isotope discrimination (Farquhar and Richards 1984). Even though this trait shows a high heritability (Condon and Richards 1992), its assessment is destructive and labour-intensive making it difficult to analyse many individuals in parallel. Stomatal conductance, a trait related to carbon isotope discrimination, is an interesting trait to select for in order to improve drought and heat tolerance.

Further, indirect traits can help to draw conclusions on genetic variation of water use associated traits. For example, stomatal conductance can be indirectly assessed by measuring leaf temperature. A low canopy temperature indicates a high stomatal conductance and can be used as an estimator for plant transpiration (Leinonen et al. 2006; Munns et al. 2010). Indeed, canopy temperature depression (the difference between the temperature of the plant and the air) has been associated with yield performance (Reynolds et al. 1999).

Considering that drought stress has extremely developmental stage-specific effects and putative traits confer only context-dependent stress tolerance (Tardieu 2012; Tardieu et al. 2018), the hope is that by continuously tracking the interplay between growth dynamics and plant response across the life cycle, one can find critical relationships that are likely to be missed by endpoint phenotyping or sporadic records. A straightforward and accurate method for estimation of plant transpiration is the gravimetric determination of transpiration and it is generally considered a valuable tool for phenotyping water characteristics due to its accuracy and simplicity and allows detailed insights into the drought stress response, which cannot be achieved under field conditions. Equipping each pot with a scale facilitates such a real-time measurement of transpiration enabling tracking of drought stress responses across all stages of the plant’s development and even genotype-specific diurnal differences in transpiration patterns (Gosa et al. 2019; Dalal et al. 2020). In combination with parallel detection of image-derived traits, as plant architecture or stay green, water use can be referred to the daily plant biomass allowing the estimation of daily transpiration efficiency (Vadez et al. 2015; Ryan et al. 2016; Halperin et al. 2017; Chenu et al. 2018; Stahl et al. 2020).

### Root architecture

In order to breed for improved abiotic stress tolerance, root morphology is a relevant target trait, as altered root system and/or roots which tap into deeper soil layers can be beneficial for plant performance under water and nutrient limited conditions and can contribute—depending on the environments—to maintaining yield and yield stability under stress conditions. But, phenotyping for root traits has a major disadvantage, especially under field conditions: root phenotyping methods are mostly invasive and labour-intensive (Trachsel et al. 2011; Perkons et al. 2014; Wu and Guo 2014). Moreover, the heterogeneity of soil characteristics in the field makes it difficult to obtain accurate phenotypic data on root properties. A faster and more accurate analysis of root system architecture becomes possible with the help of special phenotyping platforms that use transparent gels as growth medium (Hargreaves et al. 2009; Clark et al. 2011; Atkinson et al. 2019) or soil-filled rhizotrons with a transparent side for root observation (Nagel et al. 2012). Nagel et al. (2012) built a high-throughput root phenotyping platform into a greenhouse, which allowed phenotypic traits, like root system architecture, to be assessed under different environmental scenarios. Species like Arabidopsis, rapeseed, barley, wheat, and maize were grown in flat rectangular pots with one transparent side, in a fully automated fashion and both, shoot and roots were phenotyped non-invasively and thereby repeatedly. The visible portion of the root was dependent on the species, but in all cases meaningful phenotypes were collected with high repeatability depending on the trait and species (Nagel et al. 2012). While the controlled environment makes it possible to precisely measure traits like root geometry and track temporal growth responses throughout an experiment, plant biomass is likely negatively affected by the narrow pots (Poorter et al. 2012) and root system architecture in a field stand is additionally affected by the presence and competition of roots of neighbouring plants (Morris et al. 2017), which in the end makes the lab-to-field translation a challenge. Since stress conditions further decrease the accuracy of measurement and consequently the heritability of root traits (Nagel et al. 2012), the reduction of introduced noise by controlling the environment and thus generating higher achievable accuracy of measurement partially redeems CE phenotyping.

Nevertheless, as the exposure to visible light leads to phototropic responses, roots should be effectively shielded from visible light e.g. by filter material only permeable for near-infrared light (Shi et al. 2016). Magnetic resonance imaging, computed tomography and positron emission tomography are used in CE for assessing root structure (Atkinson et al. 2019; Wasson et al. 2020). On top of that, Wasson et al. 2020 present technologies to be used for non-invasive root phenotyping under field conditions, e.g. ground penetrating radar (GPR). GPR is an already established method for phenotyping coarse roots and tubers (Delgado et al. 2017; Wasson et al. 2020) but has been explored for detecting and characterizing finer roots of e.g. winter wheat, showing significant correlation between GPR signal and root parameters, depending on soil characteristics (Liu et al. 2018).

### Disease resistance

The effects of climate change also favour the spread and reproduction of pathogens, increasing disease pressure on crops (Garrett et al. 2006; Prank et al. 2019; Trebicki 2020). Crops may be exposed to new and more intense combinations of biotic stress in the future necessitating the identification of new loci that contribute to biotic and abiotic stress resistance and combinations thereof.

In order to better understand the complexity of resistance and to evaluate the efficacy of individual resistances against specific viruses or fungal isolates under realistic stress scenarios and stress combinations, artificial inoculations under CE are preferred over random natural infections. Controlled-environment phenotyping of disease resistance and tolerance is independent from the seasonality of field trials, enabling a higher frequency of testing, as well as a higher repeatability and reliability of phenotypic data compared to field conditions. In controlled environment-based approaches, adult plant resistance to leaf rust in barley (Rothwell et al. 2019) as well in wheat (Riaz et al. 2016) was phenotyped and data obtained from this study showed high correlation to results from field trials.

Automated high-throughput approaches are used for a precise and objective quantification of the degree of severity of the disease (Lück et al., 2020). Imaging techniques offer an objective and fast assessment of the plant's susceptibility to diseases (Mutka and Bart 2015). Powdery mildew symptoms of barley plants growing in large containers were quantified in an automated high-throughput manner using a hyperspectral phenotyping system (Thomas et al. 2018).

Knowledge and utilization of “omics”-data is likely to help to further understand pathogens infection strategies as well as interactions between various types of stress forms and to identify genetic components of resistances and tolerances that can be helpful for identification of genetic determinants of certain resistances (AbuQamar et al. 2016; Bindschedler et al. 2016).

## Transferability of collected data to agricultural practice is critical

Given the fact that the manifold influences of climatic changes on plant growth are influenced by a sheer myriad of lots of small effects, which in themselves may be of negligible importance, it is not surprising that effects overlap and mask each other. This makes it considerably more difficult to record and evaluate the individual effects of genes under future climate conditions. For example, genotypes that carry positive alleles for certain traits may not be identified as such because other negative effects overlap and mask the phenotype on the plant level. Moreover, its negative feedback regulations could lead to the result that alteration of specific enzymatic activities cannot be seen in the phenotype on the organ or plant level.

Many of the results on source sink relationships discovered under controlled conditions in laboratories, climatic chambers or greenhouses prove to be meaningless in practical agriculture (Fernie et al. 2020). To avoid misunderstandings, this does not mean that the results of a particular study are incorrect. Rather, one has to keep in mind that the scope of the findings is reduced only to the limited constellation of environments applied in a particular study. There is no better way to sum the G x E interaction up than the way Tardieu (2012) has expressed it: “Any trait or trait-related allele can confer drought tolerance: just design the right drought scenario”.

With this in mind future research should force the system concept and can more and more make use of the pool of data for simulations. This requires not only data from individual certain soil types, (micro) climate conditions and a limited number of genotypes, but also to explore each of these parameters factorially in order to better understand their interactions. While this was not possible in the past because research capacities were limited, digital phenotyping strategies open unprecedented options. Digital phenotyping enables the principle of zero marginal costs to be used. Accordingly, once the technology has been established, each additional phenotypic data point is available almost free of charge. While every sample/measurement used to require time and money (e.g. using wet chemical laboratory analysis), today's phenotyping using hyperspectral imaging can increase the amount of data at different scales from single cell to ecosystems many times over without exploding costs. This amount of data also provides the prerequisite for developing and operating forecast models using the latest statistical methods. The size of the data set is of critical importance. Given a well-structured and documented data matrix, state-of-the-art statistics tools allow performance predictions. While this is standard for genomic predictions and has been extremely widely applied, it has been recently successfully applied to predict yield on the basis of environmental sensitivities (Millet et al. 2019), and the integration of plant growth models is rather new territory (Technow et al. 2015). The hope is that by combining agronomic, physiological and genetic information in one model, the black box will be reduced and the predictive quality of G x E x M interactions will be improved (Cooper et al. 2021a, b).

The question arises whether the result of the modelling alone is improved by the sheer size of the data set (thousands of genotypes in thousands of environments) or whether a filigree model, in which the physiological interactions within a plant (e.g. source–sink relationships) are better mapped, contributes to the gain of knowledge. Hammer et al. (2019) have stated that this is not a contradiction in terms. Trans-scale and trans-disciplinary models from molecular level up to the scale of an ecosystem are required to deal with challenges or climate change ahead. As an example, we would like to refer to a trans-scale model approach that quantified the effect of molecular modifications on the whole plant level with its impact on photosynthesis and yield (Wu et al. 2019). It can be expected that such modelling approaches, in conjunction with artificial intelligence, based on large phenotypic, genotypic, and environmental data open completely new opportunities for targeted exploitation of bioresources (van Eeuwijk et al. 2019).

## Data management is key to knowledge discovery and innovation in breeding

Phenotypic datasets are increasingly heterogeneous and acquired from multiple sources and different scales: from the molecular phenotype (genotype and transcriptome) to whole-plant phenotypes on a single plant up to plot levels. In order to efficiently support breeders decisions by high-throughput phenotyping, appropriate data management infrastructures are of utmost importance and considered equally important for field and CE phenotyping. These need to be standardized and automated as far as possible and thereby enable informed decisions and fast ways of knowledge discovery:

- Through cross-domain data integration e.g. phenomics and transcriptomics or metabolomics (Großkinsky et al. 2017) or integration of lab and field phenomics data (Millet et al. 2019)
- Through meta-phenomics studies (Poorter et al. 2019)
- Through integration with environmental data in the G x E x M context.

Spatial correction is substantially important (Massonnet et al. 2010; Malosetti et al. 2013) and requires appropriate experimental designs (Junker et al. 2015; Cabrera‐Bosquet et al. 2016) and high resolution, well managed environmental monitoring (Neveu et al. 2019). The challenge herein is not the design of phenotyping systems (imaging sensors and other high tech equipment) but rather the design of data management systems that can handle, process, integrate and analyse the massive amounts of heterogeneous data. This is set on a range of prerequisites with respect to hardware and software capacities and features (data storage, documentation) which are supposed to meet certain guidelines referred to as the FAIR (Findable, Accessible, Interoperable, Reusable) criteria of data management (Wilkinson et al. 2016). Datasets need to be findable across decentralized and distributed storage systems through federated entry points enabling keyword-based search and browse functionalities. A key precondition for findability is accessibility of datasets which is strongly promoted through open access initiatives of funding agencies (https://www.openaire.eu/datacite) and journals (ScientificData, GigaScience). A number of general purpose FAIR data repositories nowadays exist such as e!Dal (Arend et al. 2016a), Zenodo (https://zenodo.org/), FigShare (https://figshare.com) and Dryad (http://datadryad.org). These assign unique identifiers to datasets (e.g. digital objects identifiers, DOIs) and thereby allow for referencing in open science archives. Some examples for FAIRly documented phenotypic datasets are described here: Arend et al. 2016b, Gonzalez et al. 2018, Philipp et al. 2019. Interoperability and reusability of datasets require the enrichment of data with comprehensive metadata describing biological materials, growth conditions, measurement and analysis procedures as well as protocols. For phenotypic datasets, specific recommendations are elaborated within the MIAPPE consortium (Krajewski et al. 2015; Ćwiek-Kupczyńska et al. 2016; Papoutsoglou et al. 2020), which represents a minimum information standard about plant phenotyping experiments with a checklist of metadata to document. The ISA-Tools framework (Rocca-Serra et al. 2010), as a practical implementation of the MIAPPE guidelines, represents a structured tabular format for the standardized description of experimental units, links between raw data, result data, protocols and traits and makes use of controlled vocabularies ontologies (PlantOntology Jaiswal et al. 2005; Avraham et al. 2008; CropOntology Shrestha et al. 2012; Phenotype and Trait Ontology (http://www.obofoundry.org/ontology/pato.html), UnitOntology (http://www.ontobee.org/ontology/UO). For an efficient use of FAIR datasets, interlinks between databases or repositories and (automated) analysis pipelines/routines and tools are inevitable. A recent development is the Breeding API (BrAPI, Selby et al. 2019) which enables communication between datasets from various sources using a standardized interface.

The success of these modern breeding initiatives depends on above-mentioned standardized data management which not only ensures integration and harmonization of multidimensional data but also facilitates community integration for sharing of resources in terms of datasets and beyond in terms of code and tools (Leonelli et al. 2017).

The evolution of breeding over the years correlates with the advancements in data analytics (Kuriakose et al. 2020), and respective data management is considered to be one of the main challenges for the field of phenomics (Yang et al. 2020; Coppens et al. 2017). Especially for crop growth modelling the parametrization of models with various heterogeneous datasets is important for an increase in robustness of predictions, thus having a great potential for breeders decision support in ‘prescription agriculture’ (Louarn and Song 2020; van Eeuwijk et al. 2019). In the view of these future perspectives, coordinated and structured action towards federated initiatives for phenomics data management through networks on the European (EPPN2020, https://eppn2020.plant-phenotyping.eu/ and EMPHASIS, https://emphasis.plant-phenotyping.eu/, ELIXIR, https://elixir-europe.org/) and international level (IPPN, https://www.plant-phenotyping.org/) is key to unlock the potential of phenotyping for data-driven breeding.

## Summary

Understanding how plants respond to their environment is fundamental for breeding crops that are adapted to suboptimal growing conditions and thus mitigate future risks arising from climate change. However, phenotyping for climate response traits is challenging under field conditions, as the natural occurrence and intensity of certain climate events cannot be influenced. Hence, the predicted future climate scenarios need to be simulated in CE facilities.

The phenotyping facilities mentioned in this review are examples for a wide variety of facilities that have been installed across the globe pursuing the common goal to study the plant ‘s phenotype under repeatable controlled or semi-controlled environments. The need for careful setup of growth parameters corresponding to the aim of the study, the traits and level of scale to be phenotyped are highlighted.

The recent advances in automation, imaging technologies, software solutions, and data processing support the establishment of phenotyping of plant response to certain environmental conditions in an automated, non-invasive, high-throughput, and in-depth manner.

High-throughput phenotyping platforms produce substantial amounts of data that are very valuable for plant breeding and can be used in statistical prediction models. Still, to fully exploit the potential of data derived from many independent phenotyping efforts, standardized data management infrastructures are required.
